# Gas6-Axl Signaling Induces SRF/MRTF-A Gene Transcription via MICAL2

**DOI:** 10.3390/genes14122231

**Published:** 2023-12-18

**Authors:** Mark R. Lundquist, Samie R. Jaffrey

**Affiliations:** Department of Pharmacology, Weill Medical College, Cornell University, New York, NY 10065, USA

**Keywords:** MICAL2, MRTF-A, serum response factor, Gas6, Axl

## Abstract

MICAL2 is an actin-regulatory protein that functions through redox modification of actin. Nuclear localized MICAL2 triggers the disassembly of nuclear actin, which subsequently leads to nuclear retention of the actin-binding transcriptional coregulator myocardin-related transcription factor-A (MRTF-A), which leads to the activation of serum response factor (SRF)/MRTF-A-dependent gene transcription. In this study, we show that the secreted signaling protein GAS6 (growth-arrest specific 6) and its cognate receptor Axl, a transmembrane tyrosine kinase, also induce the activation of SRF/MRTF-A and their downstream target genes. We find that serum-induced SRF/MRTF-A-dependent gene expression can be blocked, in part, by the inhibition of Axl signaling. Furthermore, we find that Gas6/Axl-induced SRF/MRTF-A-dependent transcription is dependent on MICAL2. Gas6/Axl promotes cell invasion, which is blocked by MICAL2 knockdown, suggesting that MICAL2 promotes cytoskeletal effects of the Gas6/Axl pathway. We find that Gas/6/Axl signaling promotes the nuclear localization of MICAL2, which may contribute to the ability of Gas6/SRF to augment SRF/MRTF-A-dependent gene transcription. The physiological significance of the Gas6/Axl-MICAL2 signaling pathway described here is supported by the marked gene expression correlation across a broad array of different cancers between *MICAL2* and *Axl* and *Gas6*, as well as the coexpression of these genes and the known SRF/MRTF-A target transcripts. Overall, these data reveal a new link between Gas6/Axl and SRF/MRTF-A-dependent gene transcription and link MICAL2 as a novel effector of the Gas6/Axl signaling pathway.

## 1. Introduction

The serum response factor (SRF) is a transcription factor that controls gene transcription programs linked to diverse physiological processes, most notably cellular migration [1]. SRF is affected by SRF coactivators, including ternary complex factor (TCF) and myocardin-related transcription factor A (MRTF-A) [2]. The SRF/MRTF-A complex is induced and becomes transcriptionally active when MRTF-A enters the nucleus [3]. The assembly of SRF/MRTF-A complexes results in the transcription of genes containing promoters with binding sites for the SRF/MRTF-A complex [3].

MICAL2 was shown to mediate SRF/MRTF-A-dependent gene transcription [4]. MICAL2 is an actin-regulatory protein that induces the depolymerization of nuclear actin. Monomeric actin (i.e., G-actin) is reduced in the nucleus upon MICAL2 expression, which causes the nuclear accumulation of MRTF-A [4]. Because a major function of G-actin is to bind to MRTF-A and trigger its nuclear export [5,6], the reduction in nuclear G-actin by MICAL2 leads to the nuclear accumulation of MRTF-A. As a result, MICAL2 activates SRF/MRTF-A-dependent transcription [4].

At present, the signaling pathways that utilize MICAL2-dependent control of SRF/MRTF-A signaling are not known. In this study, we set out to identify signaling pathways that utilize MICAL2. Our studies point to an unexpected role for MICAL2 in mediating the effects of the Axl signaling pathway.

## 2. Methods

### 2.1. Cell Culture

Primary antibodies used in this study are as follows: rabbit anti-GFP (1:2000; Abcam, Cambridge, UK, ab13970) and goat anti-MRTF-A (1:100; Santa Cruz Biotechnology, Santa Cruz, CA, USA, C-19). Alexa Fluor-conjugated secondary antibodies were obtained from Invitrogen (Carlsbad, CA, USA) and used at a ratio of 1:1000. F- was visualized with Phalloidin 568 (Invitrogen). F-actin was stained by incubation with Phalloidin 568 (0.3 µM) during the last 20 min of the secondary antibody incubation. MICAL2 antibody was described previously in [4]. HEK293T and A549 cells were cultured in DMEM containing 10% fetal bovine serum (Atlanta Biologicals, Flowery Branch, GA, USA) and 1X Pen-Strep (Invitrogen). Fluorescence and phase contrast images were obtained using a Nikon Eclipse TE2000-E microscope (Tokyo, Japan) using a CoolSnap HQ2 CCD camera. For each experiment, the exposure times were kept constant and in the linear range. Stacks of fluorescence images were taken and deconvoluted using AutoDeblur X (Media Cybernetics, Bethesda, MD, USA). The fluorescence intensity values (arbitrary units, AU) were obtained using Nikon’s NIS-Elements Advanced Research version 3.1 software. The *p* values were generated using Prism version 4.0. Data are presented as mean ± SEM. shRNA sequences, which were described previously in [4].

### 2.2. Nearest Neighbors Analysis

Correlations of gene expression were performed using the Cancer Cell Line Encyclopedia (https://sites.broadinstitute.org/ccle/, accessed on 25 September 2018) and the cBioPortal. The nearest neighbors analysis was performed to identify correlated gene expression [7].

### 2.3. RNA Preparation and Real-Time PCR

Total RNA was extracted from the cells using TRIzol (Invitrogen). For qRT-PCR, cDNA was prepared using Superscript III (Invitrogen) and quantified using iQ SYBR Green Supermix (Bio-rad, Hercules, CA) on a Realplex Mastercycler ep s (Eppendorf, Hamburg, Germany).

### 2.4. Luciferase Assays

Transcription mediated by SRF/TCF and SRF/MRTF-A was measured using the reporter plasmids pGL4.33 and pGL4.34, respectively (Promega, Madison, WI, USA) [4]. Luciferase activity was quantified using ONE-glo (Promega) on a Spectramax L luminometer (Molecular Devices, San Jose, CA, USA). For these experiments, cells were typically grown to 40–60% confluency and then transfected with the indicated plasmids. After 2 d, cells were trypsinized and resuspended at 5 × 10^5^ cells per mL. Then, 200 μL was plated in wells of a 96-well plate. Exactly 24 h later, cells were washed 1X with PBS and then cultured in DMEM with 0.3% FBS for serum starvation. After 24 h, wells were treated with Gas6 (as indicated) and then each well was washed with PBS. A total of 100 μL of One-Glo luciferase reagent was added, and incubated and covered with foil at 25 °C for 5 min. Luminescence was measured on a Spectramax L luminometer (Molecular Devices).

### 2.5. Axl/Gas6 Pathway Activation

The human Axl cDNA was cloned into pcDNA3.1 and used for all expression studies. Human Gas6 protein was obtained from R&D systems. GFP-MICAL2 and MICAL2 shRNA were described previously in [4].

### 2.6. Cell Migration Assay

A549 cells were plated in a serum-free medium on Transwell inserts (Corning, NY, USA), and a serum-free medium containing 0.2 µg/mL Gas6 (R&D Systems, Minneapolis, MN, USA) was added to the lower chamber. Cells were then cultured for 48 h at 37 °C. The inserts were fixed with 3.7% paraformaldehyde/PBS and the cells were detected by staining using 2% crystal violet. The number of cells that had migrated was counted in a minimum of five representative (20X) fields per insert.

## 3. Results

### 3.1. MICAL2 Expression Is Correlated with Expression Levels of SRF/MRTF-A-Dependent Genes

**Figure 1:**
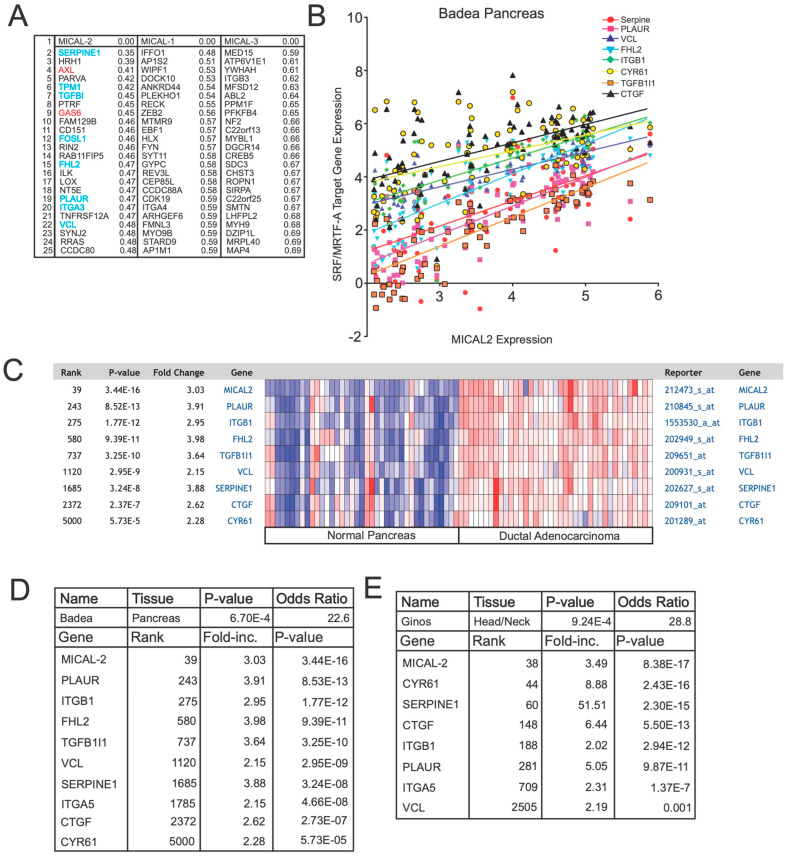
Coexpression analysis reveals a correlation between *MICAL2* and SRF/MRTF-A-regulated genes, *AXL,* and *GAS6***.** (**A**) Coexpression analysis shows that *MICAL2*, but not *MICAL1* or *MICAL3,* is coexpressed with SRF/MRTF-A-regulated transcripts as well as *AXL* and *GAS6*. In this analysis, the Cancer Cell Line Encyclopedia (CCLE) was screened for genes that correlated with *MICAL2* expression using a nearest neighbors analysis. Shown are the top 25 genes identified as coexpressed with MICAL2. Indicated are the coexpression scores, with lower numbers indicating higher coexpression. Shown in blue are genes previously documented to be regulated by SRF/MRTF-A. Shown in red are *AXL* and *GAS6*. None of these genes are among the top 25 genes that show coexpression with *MICAL1* or *MICAL3*. (**B**) *MICAL2* expression levels are positively correlated with the expression levels of SRF/MRTF-A-regulated transcripts. Shown are the transcripts that show high coexpression with *MICAL2*. As can be seen, *MICAL2* gene expression measured in the Badea pancreatic cancer gene expression dataset is positively correlated with each of the indicated SRF/MRTF-A-regulated transcripts. Expression is indicated as log_2_-fold expression. (**C**) MICAL2 expression correlates with SRF/MRTF-A-regulated genes in normal pancreas and pancreatic cancer. In normal pancreas, MICAL2 expression is low (blue) across each sample tested (each column). Overall, there is a general trend of low MICAL2 expression along with low SRF/MRTF-A-regulated gene expression in normal pancreas. In contrast, in ductal adenocarcinoma, MICAL2 expression was significantly higher, along with SRF/MRTF-A-target gene expression. “E” represents multiplication by 10 raised to the indicated power. (**D**) SRF/MRTF-A genes and MICAL2 are co-expressed as a group in the Badea pancreatic cancer database. (**E**) SRF/MRTF-A genes and MICAL2 are co-expressed as a group in the Ginos head/neck cancer database.

Although MICAL2 can induce SRF/MRTF-A complexes and subsequent gene transcription, little is known about the signaling pathways that utilize MICAL2 to mediate their effects on gene transcription. To begin to understand the cellular signaling pathways that signal via MICAL2, we screened the Cancer Cell Line Encyclopedia (CCLE) for genes that correlated with MICAL2 expressions using the nearest neighbors analysis [7]. Consistent with our earlier findings that MICAL2 regulates SRF/MRTF-A-dependent gene expression, we found that 8 of the top 25 genes that correlated most strongly with MICAL2 were also known SRF/MRTF-A target genes, including VCL, CYR61, and ITGB1 (Figure 1A,B). Each of these eight genes was previously shown to contain SRF/MRTF-A promoter elements [8]. Notably, the two MICAL2 paralogs, MICAL1 and MICAL3, show no correlation with SRF/MRTF-A-target genes (Figure 1A). This is consistent with the earlier finding that these enzymes do not activate SRF/MRTF-A-dependent gene expression [4].

The ability of MICAL2 to regulate the SRF/MRTF-A target genes was also seen when we examined a pancreatic cancer gene expression analysis (Figure 1C). Here, MICAL2 expression levels were correlated with the expression of these eight SRF/MRTF-A target genes (Figure 1C). Additionally, the expression of these genes was significantly increased as a group in cancerous tissue compared to normal tissue (Figure 1D,E).

Overall, the co-expression of these genes with MICAL2 most likely reflects the MICAL2-dependent activation of SRF/MRTF-A-dependent gene expression in various cell contexts, including cancer.

### 3.2. AXL Shows Correlated Coexpression with SRF/MRTF-A-Target Genes

However, in addition to SRF/MRTF-A target genes, both Axl, a member of the TAM family of receptor tyrosine kinases, and its cognate ligand Gas6 [9], showed marked coexpression with MICAL2 (Figure 1A). An alternate possibility is that Axl and Gas6 expression are controlled by SRF/MRTF-A. However, neither of these genes contain SRF/MRTF-A response elements and neither were identified as SRF/MRTF-A target genes in a recent genome-wide analysis of target genes [8].

We next considered the possibility that the coexpression of MICAL2 with Axl and Gas6 was an artifact due to the presence of these genes in neighboring regions on the same chromosome. In this case, coexpression could occur if all genes in a certain chromosomal neighborhood are activated due to localized epigenetic changes or duplication of a chromosomal region. However, the MICAL2, Axl, and Gas6 genes are located at distinct human chromosome locations: 11p15.3, 19q13.1, and 13q34, respectively.

Coregulated gene expression is often seen when gene products function together in the same signaling pathway. We, therefore, considered a model in which Gas6 would signal through Axl to control the MICAL2 function. MICAL2 is not currently known to be regulated by Axl, and the signaling pathways regulated by Axl are currently unclear.

**Figure 2:**
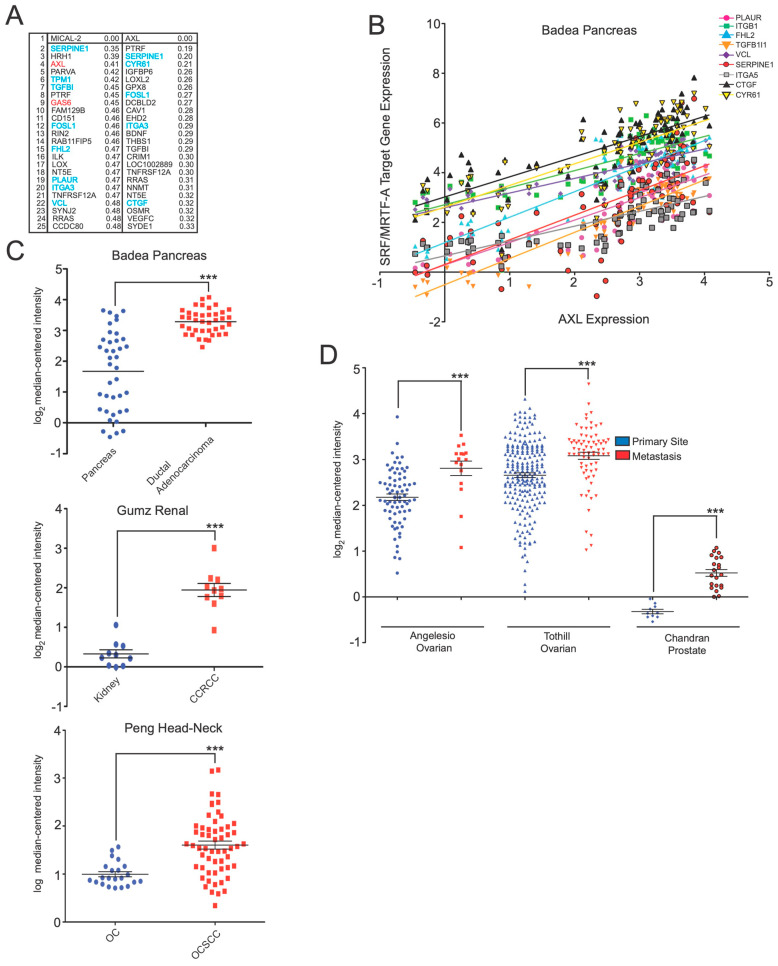
AXL expression is upregulated in diverse cancers and correlates with SRF/MRTF-A target transcripts. (**A**) Coexpression analysis shows that AXL is coexpressed with SRF/MRTF-A-regulated transcripts. In this analysis, the Cancer Cell Line Encyclopedia (CCLE) was screened for genes that correlated with *AXL* expression using a nearest neighbors analysis. Shown are the top 25 genes identified as coexpressed with *AXL*. Indicated are the coexpression scores, with lower numbers indicating higher coexpression. Shown in blue are genes previously documented to be regulated by SRF/MRTF-A. As with *MICAL2* (reproduced from Figure 1A), several coexpressed genes are SRF/MRTF-A target transcripts. Some, such as *SERPINE1* and *ITGA3*, are correlated with both *MICAL2* and *AXL*. (**B**) *AXL* expression levels are positively correlated with the expression levels of SRF/MRTF-A-regulated transcripts. Shown are the transcripts that show high coexpression with either *AXL* or *MICAL2*. As can be seen, *AXL* gene expression measured in the Badea pancreatic cancer gene expression dataset is positively correlated with each of the indicated SRF/MRTF-A-regulated transcripts. Expression is indicated as log_2_-fold expression. (**C**) *AXL* expression is elevated in pancreatic adenocarcinoma, renal cancer, and head-neck cancer. Shown are the expression data from the indicated cancer gene expression datasets. *AXL* expression is markedly upregulated in each of the indicated cancers. CCRCC, clear cell renal cell carcinoma; OC, oral cavity; OCSCC, oral cavity squamous cell carcinoma. *** *p* < 0.0005, Student’s *t*-test. (**D**) *AXL* expression is elevated in the indicated ovarian and prostate cancer datasets. *** *p* < 0.0005, Student’s *t*-test.

We reasoned that if MICAL2 is downstream of Axl, then the activation of Axl should induce SRF/MRTF-A-dependent gene expression. As a first test, we again used the Cancer Cell Line Encyclopedia but searched for genes that correlated with Axl expression using the nearest neighbors analysis approach [7]. In this case, 5 of the top 24 genes were SRF/MRTF-A targets (Figure 2A). The coregulation of Axl and SRF/MRTF-A genes is consistent with the idea that Axl regulates SRF/MRTF-A-dependent gene expression. Furthermore, we found a strong correlation between Axl expression and SRF/MRTF-A target genes (Figure 2B). This further supports the idea that Axl may regulate SRF/MRTF-A-dependent gene expression.

Notably, AXL was found as a gene correlated with MICAL2, but not vice versa. This may reflect the large number of genes that are regulated by AXL and therefore show a higher correlation than MICAL2.

Lastly, since MICAL2 is upregulated in diverse cancers [10], we also wanted to determine if Axl shows increased expression in cancer. Similarly to MICAL2, Axl is upregulated in primary tumors and further upregulated in metastases (Figure 2C,D). These data indicate that Axl-regulated pathways may also be upregulated in cancer.

### 3.3. Axl Pathway Activation Leads to Enhanced SRF/MRTF-A-Dependent Transcription

**Figure 3:**
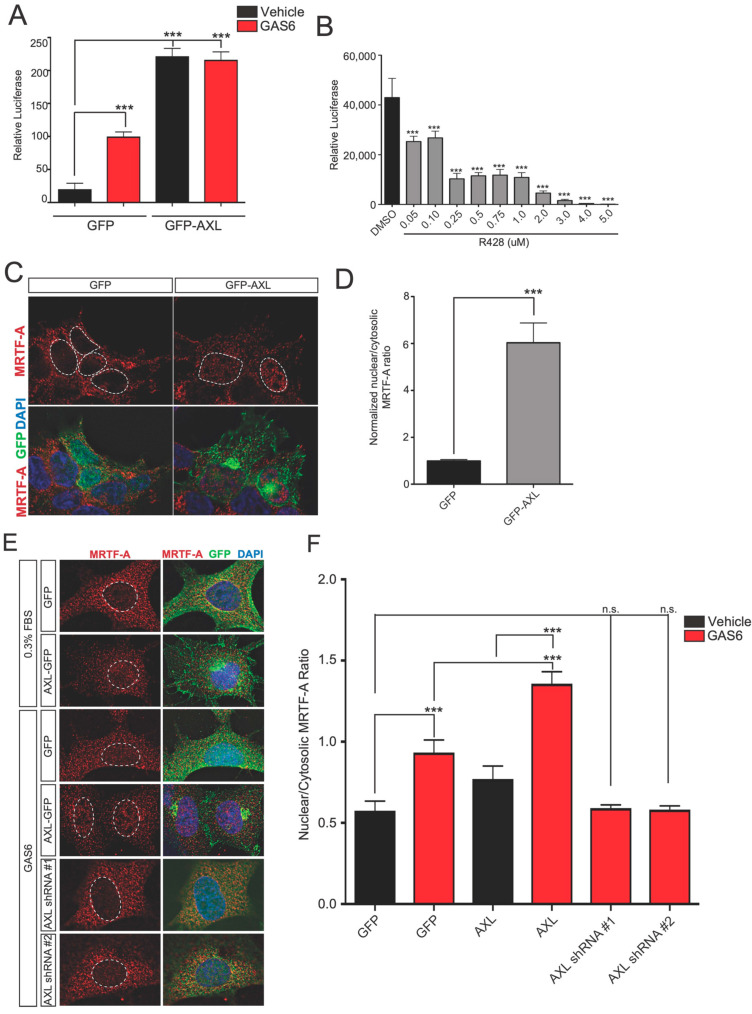
Gas6 and AXL induce the nuclear localization of MRTF-A. (**A**) Gas6 induces the SRF/MRTF-A transcriptional reporter in the absence of serum. To determine if Gas6 induces SRF/MRTF-A signaling, HEK293T cells were co-transfected with pGL4.34[luc2p/srf-re], a reporter construct expressing luciferase under the control of a SRF/MRTF-A-dependent CArG box-containing promoter. Serum-starved HEK293T (DMEM, 0.3% FBS) cells were cultured for 18 h and then treated with Gas6 (100 ng/mL) for 6 h. Statistical significance was determined by ANOVA (*** *p* < 0.005) with Dunnett multiple comparison post-test, n = 18 per condition from two biological replicates. (**B**) Axl inhibition reduces the effect of serum-induced SRF/MRTF-A reporter activation. HEK293T cells were cultured in 10% FBS for 18 h and subsequently treated with the indicated concentrations of R428, an Axl receptor inhibitor for 6 h. Statistical significance was determined by ANOVA (*** *p* < 0.005) using Dunnett multiple comparison post-test, n = 18 per condition from two biological replicates. (**C**) GFP-AXL expression induces nuclear localization of MRTF-A. HEK293 cells were infected with lentivirus expressing either GFP or GFP-AXL for 24 h. Dotted white lines show the outline of the nuclei, based on DAPI staining. (**D**) Quantification of results in (**C**). The average intensity of MRTF-A immunofluorescence was quantified in the nucleus and the cytoplasm. These values were used to calculate the ratio. This ratio is useful for reducing variability due to MRTF-A expression levels between cells. GFP-AXL increases the nucleus:cytoplasmic ratio of MRTF-A by ~6-fold. *** *p* < 0.0005, Student’s *t*-test, n ≥ 30 per condition from two biological replicates. (**E**) AXL is required for Gas6-induced nuclear localization of MRTF-A in HEK293 cells. Serum-starved A549 (DMEM, 0.3% FBS) cells were cultured for 18 h and then treated with Gas6 (100 ng/mL) for 6 h. GFP-AXL (green) expression results in increased levels of nuclear MRTF-A and this was further increased by the application of Gas6. Baseline levels of nuclear MRTF-A are determined based on the levels seen in GFP-expressing cells. Gas6 caused an increase in nuclear MRTF-A localization, which was blocked by lentiviral expression (indicated by GFP fluorescence) of either of two different Axl-specific shRNA. Nuclei are outlined by dotted white lines in the representative images shown. (**F**) Quantification of results in (**E**). *** *p* < 0.0005, statistical significance was determined by ANOVA (*** *p* < 0.0001) using Dunnett multiple comparison post-test, n ≥ 30 per condition from two biological replicates.

To further test the idea that Axl regulates SRF/MRTF-A-dependent gene transcription, we used a reporter plasmid for SRF/MRTF-A encoding luciferase downstream of the CArG [CC(A/T)_6_GG] sequence element [11]. This motif confers transcriptional responses to SRF/MRTF-A [11]. In these experiments, the reporter was transfected into HEK293 cells cultured in media containing minimal serum (0.3% FBS) to ensure a low activity of the reporter. In GFP-expressing cells, luciferase expression was minimal in vehicle-treated cells but was induced following treatment with Gas6 (Figure 3A). However, the expression of GFP-Axl resulted in a ~10-fold increase in luciferase expression; however, the treatment of GFP-Axl-expressing cells with Gas6 did not further induce an expression of the reporter, suggesting that the expression of GFP-Axl saturated the activation of this signaling pathway. (Figure 3A). Thus, in the absence of serum stimulation, Axl expression induces SRF/MRTF-A-mediated transcription.

Since Gas6 is a ligand of Axl, we next examined whether the application of Gas6 induces the SRF/MRTF-A reporter. The treatment of HEK293 cells with Gas6 (300 ng/mL) for 4 h caused a ~five-fold increase in luciferase expression relative to the vehicle (Figure 3A). This suggests that Gas6-Axl signaling leads to SRF/MRTF-A-dependent gene transcription.

Since Gas6 is a constituent of serum [12], we asked if Gas6/Axl signaling contributes to the documented ability of serum to induce the activation of SRF/MRTF-A-dependent transcription [13]. We, therefore, examined the effect of pharmacologic Axl inhibition on the SRF/MRTF-A reporter in HEK293 cells cultured in media containing 10% FBS. In vehicle-treated cells, luciferase activity was high (Figure 3B). However, the treatment of cells with the Axl-specific inhibitor R428 (Figure 3B) [14] significantly reduced the activity of the SRF/MRTF-A reporter in a dose-dependent manner. These data indicate that serum-induced SRF/MRTF-A activity may be mediated, in part, by the activation of Axl-dependent signaling.

Overall, these data support the idea that Axl and Gas6 can activate SRF/MRTF-A-dependent gene transcription.

### 3.4. Gas6 and Axl Promote the Nuclear Localization of MRTF-A

We next wanted to determine if MICAL2 may mediate the effects of Gas6/Axl signaling on SRF/MRTF-A-mediated transcription. MICAL2 induces the activation of SRF/MRTF-A-dependent transcription by inducing the nuclear localization of MRTF-A. To determine if this mechanism could mediate the effects of Gas6/Axl signaling, we monitored MRTF-A localization in HEK293 cells transfected with GFP or GFP-Axl to increase Axl signaling. In these experiments, cells were cultured in minimal serum (0.3% FBS). In GFP-expressing cells, minimal nuclear localization of endogenous MRTF-A was detected by immunofluorescence. However, in GFP-Axl expressing cells, the nuclear localization of MRTF-A was readily detected (Figure 3C,D). Thus, increased Axl expression leads to nuclear accumulation of MRTF-A.

**Figure 4:**
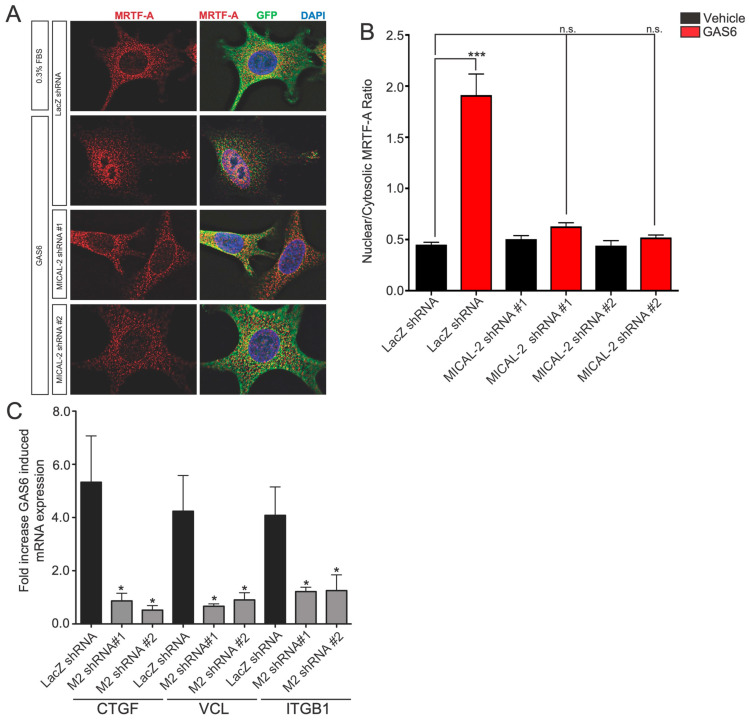
Gas6-induced nuclear localization of MRTF-A and Gas6-mediated induction of SRF/MRTF-A target genes are mediated by MICAL2. (**A**) MICAL2 is required for Gas6-induced nuclear localization of MRTF-A in HEK293 cells. Serum-starved HEK293T (DMEM, 0.3% FBS) cells were cultured for 18 h and then treated with Gas6 (100 ng/mL) for 6 h. A549 cells infected with lentivirus expressing either control shRNA (lacZ) or MICAL2-specific shRNA. Infected cells express GFP (green). Shown are the representative cells from immunofluorescence staining experiments from two independent biological replicates with the indicated antibodies. Gas6 treatment resulted in increased levels of nuclear MRTF-A, but this effect was blocked in cells expressing either MICAL2-specific shRNA. (**B**) Quantification of results in (**A**). *** *p* < 0.0005, statistical significance determined by ANOVA (*** *p* < 0.0001) using Dunnett multiple comparison post-test, n ≥ 30 per condition from two biological replicates. n.s., not significant. (**C**) Gas6-mediated induction of SRF/MRTF-A-regulated transcripts is dependent on MICAL2. The indicated that SRF/MRTF-A-target transcript (CTGF, VCL, or ITGB1) was measured in A549 cells using qRT-PCR following treatment with Gas6, and the fold increase relative to vehicle-treated cells is indicated. Expression of each gene is markedly increased following Gas6 treatment. This effect is blocked in cells infected with lentivirus expressing either of the two MICAL2-specific shRNA, but not control shRNA. * *p* < 0.05, statistical significance was determined by ANOVA using Dunnett multiple comparison post-test, n ≥ 3 per gene.

We next wanted to assess whether the connection between Axl/Gas6 and SRF/MRTF-A signaling can be seen in other cell lines. We, therefore, used the A549 lung cancer cell line, which is often used to study Gas6 signaling. The treatment of A549 cells with Gas6 (300 ng/mL, 1 h) induced the nuclear localization of MRTF-A, which was not observed in vehicle-treated cells (Figure 3E,F). The effects of Gas6 were mediated by binding to Axl, as Axl-specific shRNA blocked this effect (Figure 3E,F). The control cells (expressing GFP) (Figure 3E,F) or LacZ shRNA expression (Figure 4A,B) both showed much less nuclear localization of MRTF-A. It should be noted that the effects of GFP-Axl in A549 cells are less pronounced than in HEK293T cells in all replicates (see Figure 3C,D). The basis for this is unclear, but it might reflect reduced Axl signaling efficiency in A549 cells. Collectively, these data indicate that Gas6/Axl signaling leads to increased nuclear accumulation of MRTF-A in two different cell lines.

### 3.5. MICAL2 Promotes Gas6-Dependent MRTF-A Nuclear Localization and Gas6-Dependent Transcription of SRF/MRTF-A Target Genes

We next directly tested if MICAL2 is downstream of Gas6/Axl signaling to regulate SRF/MRTF-A. As a first test, we examined whether MICAL2 mediates the nuclear localization of MRTF-A induced by Gas6/Axl signaling. To test this, we treated A549 cells with control (LacZ) or MICAL2-specific shRNA [4] and monitored the nuclear localization after treatment with Gas6. In LacZ shRNA-expressing A549 cells treated with Gas6, MRTF-A localization was predominantly nuclear (Figure 4A,B). However, cells that expressed either of the two MICAL2-specific shRNA showed markedly reduced Gas6-induced MRTF-A nuclear localization (Figure 4A,B). These data indicate that MICAL2 promotes the ability of Gas6/Axl signaling to induce SRF/MRTF-A-dependent nuclear localization of MRTF-A.

We next monitored the role of MICAL2 in SRF/MRTF-A-dependent gene transcription induced by Gas6/Axl signaling. The application of Gas6 (300 ng/mL, 3 h) to A549 cells cultured in low (0.3%) serum induced known SRF/MRTF-A-regulated genes such as *CTGF*, *VCL*, and *ITGB1* (Figure 4C). This effect was significantly reduced in cells expressing either of the two MICAL2-specific shRNA (Figure 4C). Thus, the Gas6-induced expression of these target genes is mediated by MICAL2.

Overall, these data indicate that MICAL2 is needed for the Axl/Gas6-mediated nuclear localization of MRTF-A and Axl/Gas6-dependent regulation of SRF/MRTF-A target genes.

### 3.6. Gas6 Promotes Nuclear Localization of MICAL2

**Figure 5:**
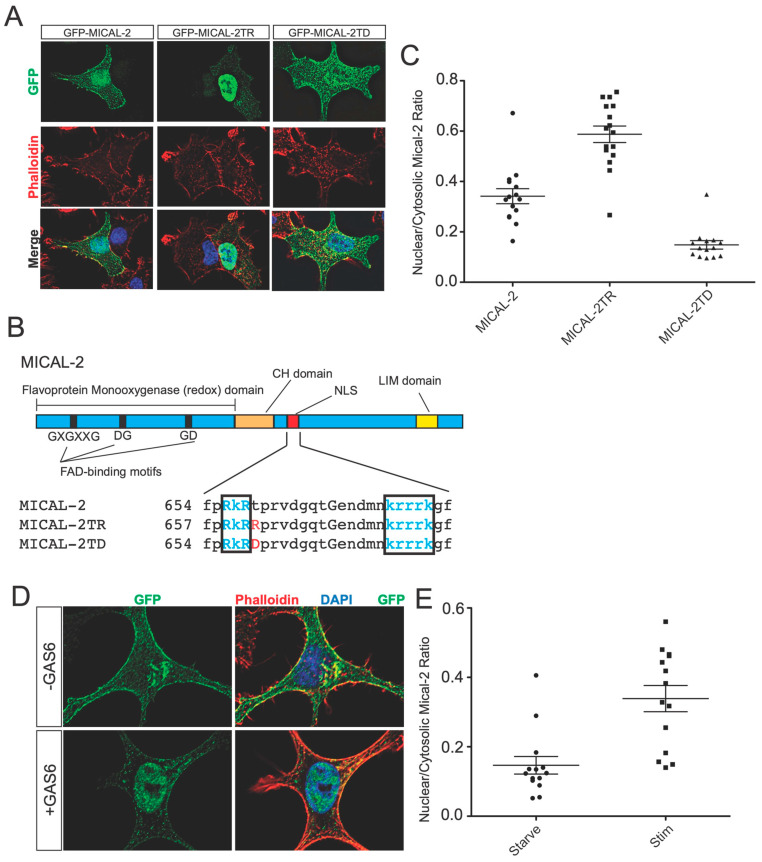
Gas6 increases the nuclear localization of MICAL2. (**A**) Validation of a nuclear localization element in MICAL2 and identification of mutants that affect the nuclear localization of MICAL2. GFP-MICAL2 was expressed in A549 cells, and the localization was detected by green fluorescence. F-actin was detected by staining with Phalloidin-Alexa 594. Shown are the representative cells from two independent biological experiments. In cells expressing GFP-MICAL2, localization was enriched in the nucleus with some protein readily detected in the cytosol. However, expression of a mutant in which a potentially regulated threonine (see Figure 5B) is mutated to arginine (GFP-MICAL2TR) resulted in green fluorescence primarily restricted to the nucleus. In contrast, a phosphomimetic mutant of the same residue (GFP-MICAL2TD) shows increased cytosolic localization relative to the wild type. (**B**) MICAL2 and -3 each contain a putative bipartite NLS. The domain structure of MICAL2 is indicated above. The enzymatic domain contains a GXGXXG, aspartate-glycine (DG), and a glycine-aspartate (GD) motif, which is the characteristic FAD-binding motif. The F-actin binding CH domain and structural LIM domain are indicated. The red box is the NLS domain in MICAL2. Comparison of the primary sequences of MICAL1, 2, and 3 shows a potential bipartite NLS in MICAL2 and MICAL3 that is not found in MICAL1. Shown in blue letters are the amino acids that constitute the NLS in MICAL2 and -3. Shown in red are the amino acids that are mutated in the M2NLSMut and M3NLSMut. Uppercase letters are amino acids conserved in MICAL-1, -2, and -3. (**C**) Quantification of results in (**A**). (**D**) Gas6 induces nuclear localization of MICAL2. A549 cells were cultured in low serum (0.3% FBS) and infected with lentivirus expressing GFP-MICAL2 (green). Shown is a representative example of cells from two independent biological replicates. F-actin was labeled with phalloidin-Alexa 594 (red). Nuclei are labeled with DAPI (blue). Treatment with Gas6 (100 ng/mL, 30 min) resulted in increased levels of nuclear localized MICAL2. (**E**) Quantification of results in (**D**). “Starve” refers to 0.3% FBS, and “Stim” refers to 0.3% FBS plus Gas6 (100 ng/mL).

We next sought to understand how Gas6/Axl might affect MICAL2 to induce SRF/MRTF-A-dependent gene transcription. We previously showed that endogenous MICAL2 is primarily nuclear in HEK293, and this localization was unaffected by serum [4]. Additionally, other cell lines, such as COS7 and HeLa, showed predominantly nuclear staining. However, we noticed that in some cell lines, such as A549 cells, a portion of GFP-MICAL2 was detectable in the cytosol (Figure 5A). GFP-MICAL2 was used to assess MICAL2 localization in these cells due to nonspecific background labeling using MICAL2 antibodies.

Unlike HEK293 cells which exhibit primarily nuclear labeling of GFP-MICAL2, GFP-MICAL2 is detected in both the cytoplasm and nucleus of A549 cells, with more prominent labeling in the nucleus (Figure 5A). We further confirmed the specificity of this localization by mutating a putative nuclear-localization site (Figure 5B) that we identified previously [4]. Increasing the positive charge of a nuclear-localization element is associated with enhanced nuclear localization function [15]. We, therefore, introduced a T659R mutation, which increased the nuclear localization. In contrast, increasing the negative charge with a phosphomimetic T659D mutation decreased the nuclear localization. Overall, these experiments confirm immunofluorescence labeling and that the nuclear localization of MICAL2 is derived from an internal nuclear-localization element.

Since MICAL2 appears to function in the nucleus, pathways that increase its nuclear localization might augment its nuclear functions. We therefore asked if an increase in nuclear localization of MICAL2 is induced by Gas6/Axl signaling. To test this, we applied Gas6 to A549 cells. Within 30 min of Gas6 application, MICAL2 relocalized to the nucleus (Figure 5D,E). F-actin in the nuclei of these cells was present as relatively small puncta and appeared to be reduced in number after Gas6 treatment (Figure 5D), consistent with MICAL2’s ability to depolymerize nuclear F-actin [4]. Notably, nuclear F-actin in A549 cells exhibited a different morphology than nuclear F-actin in HEK293T cells reported previously in [4]. Overall, these data demonstrate that Gas6 induces the nuclear localization of MICAL2, which may enhance its ability to regulate nuclear actin and subsequently SRF/MRTF-A-dependent gene expression.

Although MICAL2 is capable of activating SRF/MRTF-A-dependent gene transcription, the mechanism by which MICAL2 is activated to initiate SRF/MRTF-A-dependent gene transcription is not clear. Although the nuclear translocation of MICAL2 occurs in A549 cells, MICAL2 is constitutively nuclear in other cell types, including HEK293 cells [4]. Thus, additional mechanisms might be used in other cell types.

### 3.7. MICAL2 Is Required for the Gas6-Induced Cellular Migration

**Figure 6:**
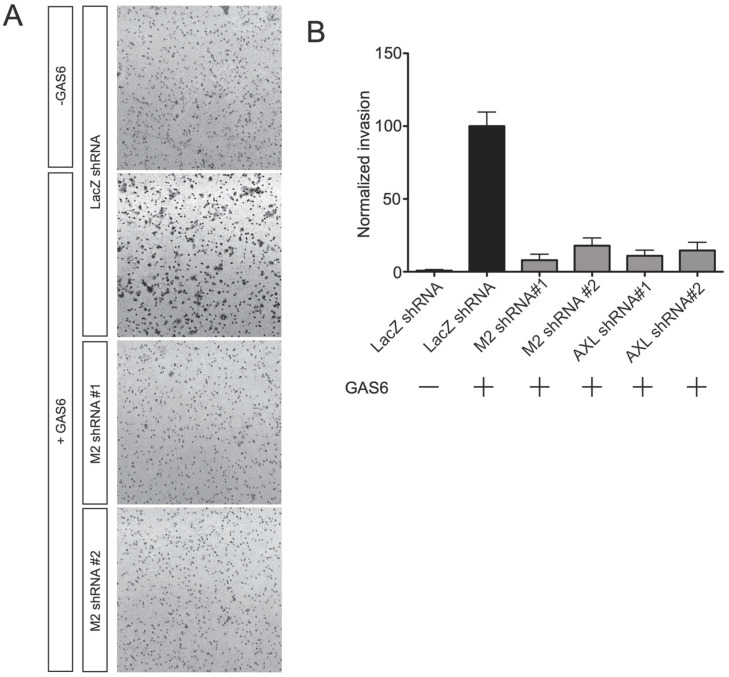
MICAL2 is required for Gas6-induced migration of A549 cells. (**A**) A549 cells were plated on Transwell inserts and migration through the membrane was measured 48 h after adding Gas6-containing media in the lower chamber. Cells were detected by staining with crystal violet. In cells expressing the control (LacZ) shRNA, Gas6 caused a marked increase in cell migration. However, this effect was not seen in cells expressing either of the two MICAL2-specific shRNA (M2 shRNA). Shown are representative results from three independent replicates. (**B**) Quantification of migration in (**A**). n ≥ 40 20X fields of view per condition.

Gas6/Axl regulates pathways linked to cellular migration and chemotaxis [16,17]. To determine if these aspects of Gas6/Axl signaling are mediated by MICAL2, we monitored the cellular migration of A549 cells. These cells have previously been used to study Gas6/Axl-induced cellular invasion [18,19]. As expected, the addition of Gas6 induced migration through the matrigel to the other side of the transwell. As previously shown **in** [19], this effect was dependent on Axl as it was blocked by either of the two Axl-specific shRNA (Figure 6A,B). The Gas6-induced cellular migration was also blocked by MICAL2-specific shRNA (Figure 6A,B). These data suggest that Gas6/Axl-dependent cellular migration is mediated by MICAL2.

## 4. Discussion

Gas6/Axl signaling has emerged as a major regulator of diverse cellular processes which involve cellular migration [19]. However, the mechanism by which Gas6/Axl mediates these effects has remained poorly understood. Our work links the effects of Gas6/Axl to SRF/MRTF-A-dependent gene expression, which has well-known effects on cellular migration by influencing specific patterns of gene expression. Moreover, these effects are promoted by MICAL2, a recently identified regulator of SRF/MRTF-A-dependent gene expression [4]. The blockade of MICAL2 prevents the migration effects induced by Gas6, implicating the SRF/MRTF-A pathway as a major mediator of the effects of Gas6/Axl.

The connection between Gas6/Axl was initially identified based on gene correlations between these genes and SRF/MRTF-A target genes, as well as MICAL2. Although the connection between MICAL2 and SRF/MRTF-A was previously described based on studies showing that MICAL2 regulates nuclear G-actin [4], the correlation studies presented here are particularly powerful because they demonstrate the relationship between these pathways in diverse cellular contexts and in disease states. The correlation points to the disease relevance of Gas6/Axl, MICAL2, and subsequent regulation of SRF/MRTF-A gene expression. Although these gene correlations were observed based on an analysis of cancer datasets, it will be important to determine if Axl also regulates MICAL2 and SRF/MRTF-A in development and other physiological processes.

Although much of the focus of therapeutic targeting of Axl in cancer is on receptor inhibitors, these studies point to the potential value of inhibiting MICAL2 or SRF/MRTF-A signaling to block the effects of elevated Axl in cancer. These compounds include CCG-1423, an inhibitor of SRF/MRTF-A signaling [20], which we previously found to bind to MICAL2 as well. However, CCG-1423 appears to show complex binding properties and other intracellular targets, including MRTF-A itself [21]. Thus this compound and newer high affinity/specificity versions [22] might be useful to limit Axl signaling in cancer.

Notably, we observed that Gas6/Axl signaling is associated with increased nuclear localization of MICAL2. Our previous studies using HEK293T and COS7 cells showed that MICAL2 was predominantly nuclear [4]. However, subsequent analysis presented here shows that other cell lines also express MICAL2 in the cytoplasm. In this case, Gas6/Axl-induced translocation of MICAL2 into the nucleus could be an important part of the mechanism that leads to the activation of the nuclear activity of MICAL2. However, additional mechanisms are likely to be operational since MICAL2 is already nuclear in many other cell lines. Future studies of MICAL2 regulation, such as post-translational modifications, might reveal additional mechanisms that could contribute to the ability of Gas6/Axl to activate MICAL2-dependent signaling. Additionally, it will be interesting to determine if cytoplasmic MICAL2 can influence cytoplasmic F-actin pathways.

In summary, this study suggests that MICAL2 is an effector of Gas6/Axl signaling and reveals a role of Gas6/Axl in regulating SRF/MRTF-A-dependent gene transcription. Overall, these studies markedly expand our understanding of this cancer-relevant pathway and suggest new avenues for therapeutic intervention of Axl-driven cancer.

## Data Availability

No new data were created or analyzed in this study. Data sharing is not applicable to this article.
